# Sensitive detection of tamsulosin hydrochloride based on dual-emission ratiometric fluorescence probe consisting of amine-carbon quantum dots and rhodamine B

**DOI:** 10.1038/s41598-021-00363-x

**Published:** 2021-10-21

**Authors:** Aida Mousavi, Rouholah Zare-Dorabei, Seyed Hossein Mosavi

**Affiliations:** Research Laboratory of Spectrometry & Micro and Nano Extraction, Department of Chemistry, Iran University of Science and Technology, 16846-13114 Tehran, Iran

**Keywords:** Analytical chemistry, Materials chemistry, Nanoscience and technology, Nanomedicine, Diagnostic devices

## Abstract

In this work, amine-carbon quantum dots (CQDs)/rhodamine B (RhB) ratiometric fluorescent (RF) sensor was employed for effective and selective determination of tamsulosin hydrochloride (TMS) based on a dual-emission fluorescence system. Although the function of amine-CQDs is to transfer the specific interaction between TMS and sensor into detectable fluorescence (FL) signals, RhB as a reference unit has been employed to omit internal and external effects. The FL signal was quenched by adding the TMS at 442 nm; nevertheless, it did not change at 569 nm. The material characterization and investigation of the sensing mechanism were done. The optimization of pH, the volumetric ratio of CQDs to RhB, and interaction time parameters were carried out by the one-variable-at-a-time (OVAT) method. The quantitative analysis of the concentration of TMS for this RF sensor in a linear range of 0.446–7.083 μg mL^−1^ (1.091–17.338 μM) was obtained (R^2^ = 0.9969, n = 3) under optimum conditions. The limit of detection and quantitation values were estimated to be 0.033 μg mL^−1^ (0.081 μM) and 0.109 μg mL^−1^ (0.267 μM), respectively. The repeatability of intra-day and inter-day were less than one percent. This inexpensive RF probe was well applied to determine TMS in biological fluids, and acceptable achievements were obtained.

## Introduction

Tamsulosin hydrochloride (TMS) 5-[(2R)-2-[[2-(2-Ethoxyphenoxy)ethyl]amino]-propyl]-2-methoxybenzenesulfonamide hydrochloride, is a class of alpha-1 (α1) adrenergic receptor antagonist family, which is used for monotherapy benign prostate hyperplastic (BPH)^1,2^. These antagonist agents block α1A-receptors in the bladder’s neck, and the prostate results in appeasing symptoms of BPH by relaxing the smooth muscle there^3^. Up to now, several analytical techniques have been reported for recognition of TMS, such as high-performance liquid chromatography (HPLC)^4^, liquid chromatography-MS^5^, capillary electrophoresis (CE)^6,7^, electrochemical methods (EC)^8^, UV–vis spectrophotometry^9^, fluorescence spectroscopy (FL)^10^. Chromatographic methods, despite the excellent measurement advantages in trace concentrations, require expensive equipment like CE assay, and toxic solvents; also, EC methods have weak repeatability^11,12^. Thus, developing a rapid and inexpensive method with high accuracy is needed to determine TMS in biological fluids.

Fluorescent probes have recently become one of the most popular techniques for chemo/bioimaging and chemo/biosensing because the FL method is a low-cost, high-selectivity assay technique compared to radioactive tracers used for biological assays. The ratiometric fluorescent (RF) probes are one of the fluorescent sensors, which have attracted considerable attention because these sensors have higher sensitivity and accuracy than single-signal sensors. Single-signal probes are not accurate enough to determine owing to the effects of FL fluctuations and intrinsic background FL, while dual-signal RF probes have a self-calibration to eliminate background interferences and environmental effects, so this is an accurate and reliable method of measuring biological and pharmaceutical samples. In general, an RF probe should be made of two different fluorophores in a sensing system^12–19^.

Fluorescent carbon quantum dots (CQDs), as a prominent member of carbon-containing nanomaterials, which researchers take an interest in CQDs over the past decade due to their unequaled properties such as excellent water dispersibility, non-toxicity, superior biocompatibility, easy preparation, inexpensive, resistance to photobleaching and optical stability^20–27^. These extraordinary properties of CQDs have turned them into ideal materials in biosensors^28^, light-emitting devices^29^, drug delivery^30^, and imaging^31^. The quasi-spherical CQDs nanoparticles are less than 10 nm in size and are composed of the core and the shell. The core is constituted of sp^2^-hybridized carbon clusters, and the shell contains sp^3^-hybridized carbons, which there are different functional groups on their surface^24,32^.

Herein, amine-CQDs with intense blue FL were synthesized by a one-step hydrothermal method from precursors of EDA and CA. Synthetic amine-CQDs have good water dispersibility, high luminescence, and galore functional groups. In this study, we reported CQDs/RhB as a novel RF probe for the TMS determination. Components amine-CQDs and RhB make up this RF sensor; amine-CQDs act as a receptor and reporter element that has been employed to determine the TMS, while RhB has been used as a reference element due to its optical stability, attractive photophysical properties, and high quantum yield, which reduces interferences caused by environmental factors^33,34^. In the FL spectra of this sensor, two emission peaks are observed, the peak of amine-CQDs appearing at 442 nm and the peak of RhB at 569 nm. By increasing TMS concentration to the RF sensor, the emission of amine-CQDs was turned-off while the emission of RhB remained constant. In order to investigate the practical application of this probe in real biofluids samples, it was employed to determine the TMS. This approach of measuring TMS by amine-CQDs/RhB RF probe is not only a quick, facile, and inexpensive method, but also due to the use of two types of fluorophores and having a dual emission signal, it is able to eliminate the annoying effects of the system; thus it is a sensitive and accurate method. So far, no literature has been reported for the detection of TMS by dual emission RF probes.

## Results

### Characterization

**Figure 1:**
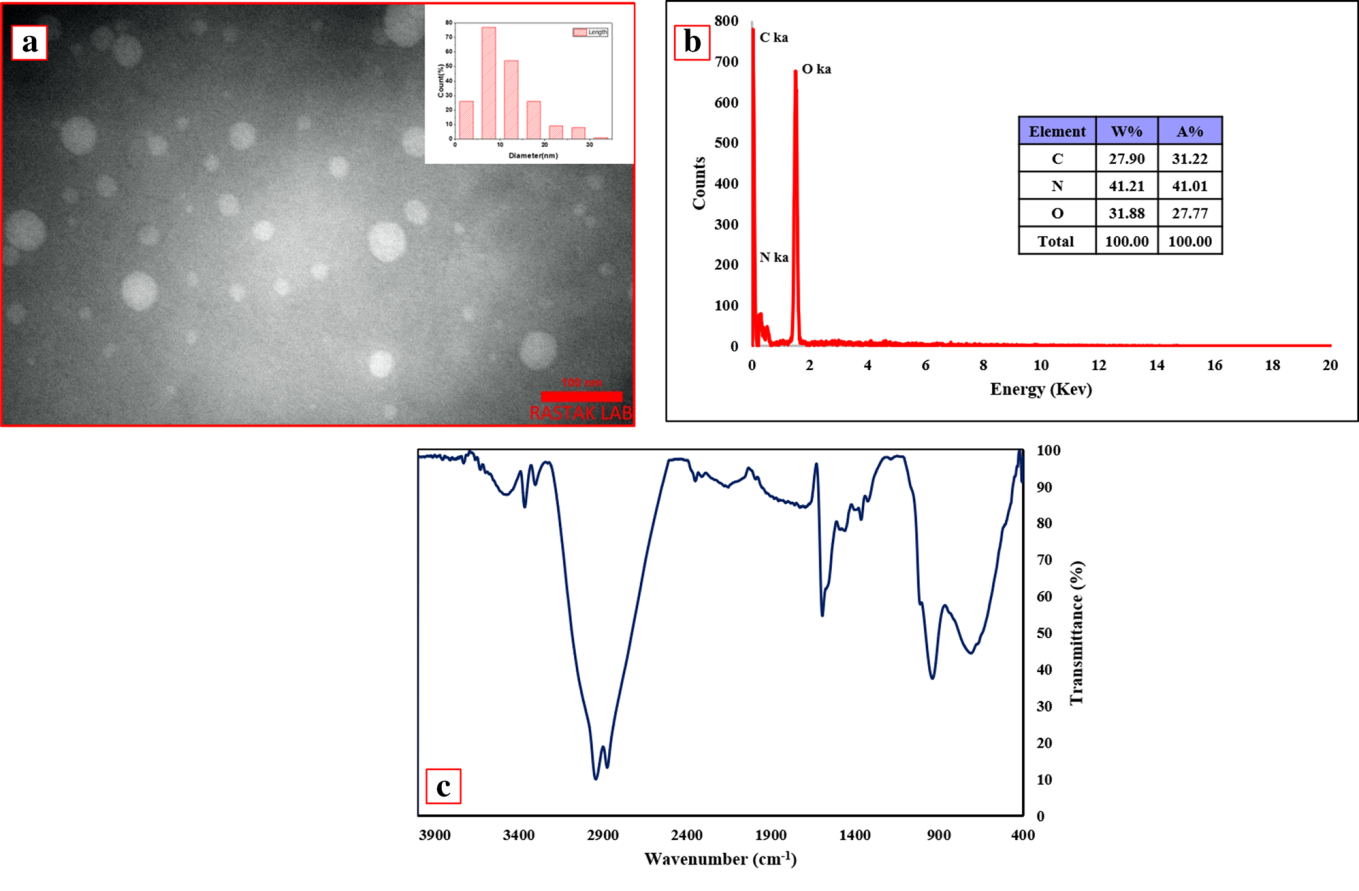
(**a**) TEM image and particle size distribution, (**b**) EDS analysis, and (**c**) FT-IR spectrum of amine-CQDs.

The morphology of the fabricated amine-CQDs was explained by transmission electron microscopy (TEM). The TEM image displays that CQDs have a spherical shape, and the mean diameter size of these nanoparticles was estimated to be roughly 7.5 nm (Fig. 1a). To identify the elements of prepared amine-CQDs, EDS analysis was carried out. As shown in Fig. 1b, the elements carbon, oxygen, and nitrogen were found. The Fourier transform infrared (FT-IR) spectrum of amine-CQDs is demonstrated in Fig. 1c. The band at 3473 cm^−1^ belongs to the stretching vibration of OH. Two peaks at 3364 cm^−1^ and 3301 cm^−1^ are ascribed to the NH asymmetric and symmetric stretching vibrations, respectively. The asymmetric and symmetric stretching vibrations of CH emerged at 2941 cm^−1^ and 2873 cm^−1^, respectively. Further, the absorption peak at 2154 cm^−1^ corresponds to C = C vibration. The vibrations at 1594 cm^−1^ and 1458 cm^−1^ are attributed to C = O stretching and NH bending vibrations, respectively. The absorption band at 1363 cm^−1^ is assigned to C–N vibration, and the peak at 710 cm^−1^ is ascribed to the out-of-plane NH bending vibration. Furthermore, The FT-IR spectra of RhB and TMS are reported in Figure S1.

The optical properties of amine-CQDs were investigated by the spectra of UV–Vis and Excitation-Emission photoluminescence (PL). In Figure S2a, two typical absorption peaks are located at 245 nm and 340 nm, which are attributed to the π → π* transition of C=C and n → π* transition of C=O, respectively. The highest emission intensity is observed under excitation at a wavelength of 365 nm (Figure S2b). The stability of amine-CQDs was investigated with absorption and FL spectra. The photostability of amine-CQDs was assessed after two months of storage; as shown in Figure S3, no noticeable change in FL and UV–Vis intensities is observed. According to Figure S4, the FL intensity of amine-CQDs can remain stable during UV irradiation for thirteen minutes.

### Optimization of experimental parameters

**Figure 2:**
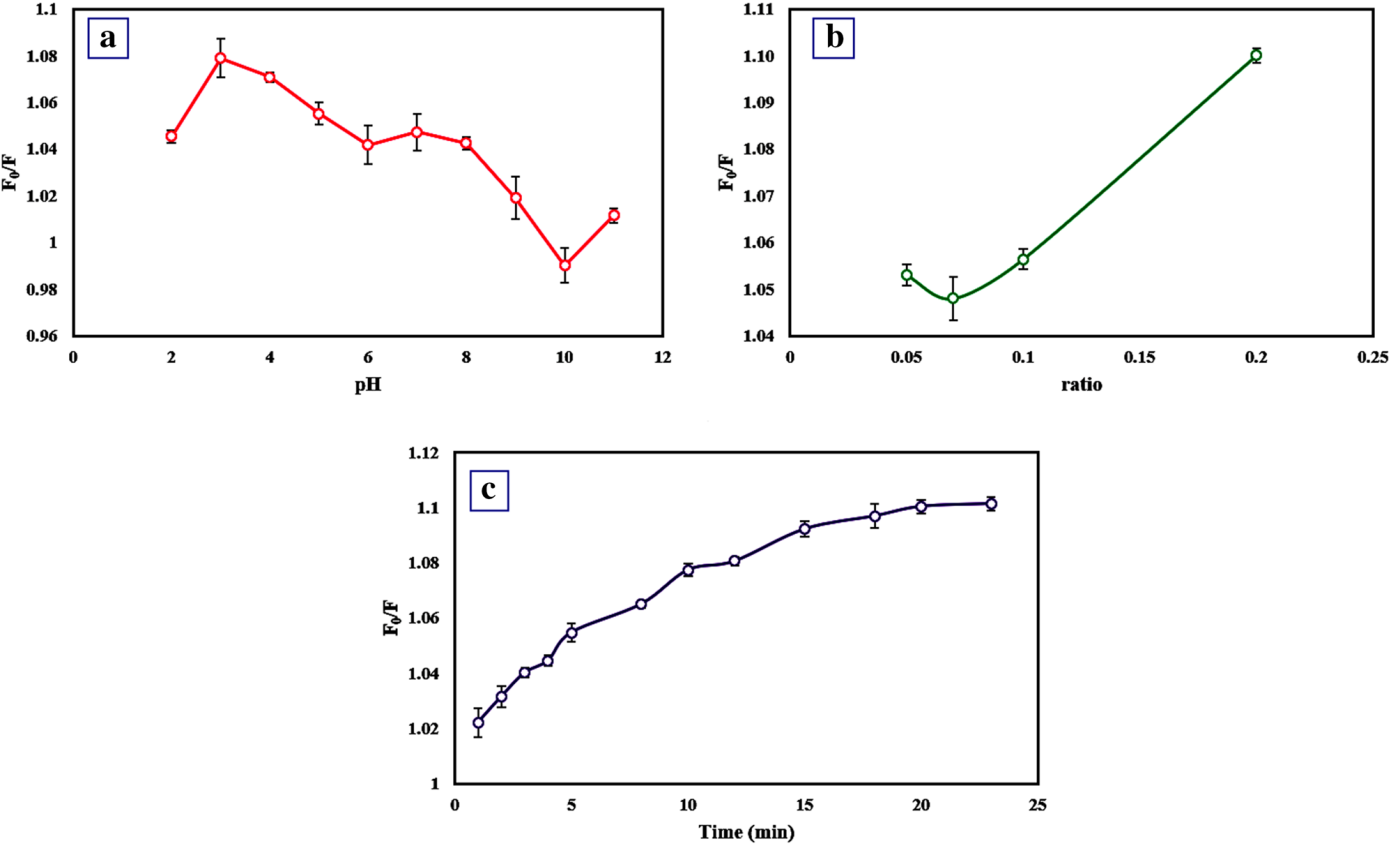
Effect of (**a**) pH (pH = 2–11, and C_TMS_ = 4.2 μg mL^−1^), (**b**) volume ratio (ratio = 0.05–0.2, pH = 3, and C_TMS_ = 4.2 μg mL^−1^), and (**c**) interaction time (t = 0–23 min, pH = 3, ratio = 0.2, and C_TMS_ = 4.2 μg mL^−1^). Error bars represent the standard deviation from three repetitions.

In order to achieve an excellent response for TMS determination, consequential factors such as pH, the ratio of fluorophores, and interaction time were optimized for the RF sensor. The pH effect on the F_0_/F value (F_0_ and F are the FL intensities ratio of the RF sensor in the absence and presence of TMS, where F_0_ = (F_442_/F_569_)_0_ and F = (F_442_/F_569_)) was examined by tuning the B-R buffer from 2 to 11. As shown in Fig. 2a, the F_0_/F value increases by moving to more acidic pHs, while other parameters are considered constant. So, the maximum value is related to pH = 3, which is selected as the optimum value because the functional group on the CQDs can be protonated by catching H^+^ at acidic media; thus, the active sites of CQDs increase. The second parameter was the volumetric ratio of CQDs to RhB, where pH and other parameters were kept constant. By enhancing the volumetric ratio, the values of F_0_/F were raised, so according to Fig. 2b, the volumetric ratio of 0.2 was chosen as the optimal point because by increasing the concentration of RhB, its interaction with functional groups of CQDs enhances, so lead to reduce the active sites of CQDs, consequently bring about decrease the interaction between CQDs and TMS. The interaction time as the third parameter was optimized, while pH = 3, the volumetric ratio of 0.2, and other items were maintained constant. As illustrated in Fig. 2c, by raising time up to 15 min, the F_0_/F value goes up with a steep slope, and then it becomes approximately invariant. Fifteen minutes was the best interaction time because it takes more time for the analytes to reach the surface of the sensor; for this reason, the interaction time has increased.

### Quenching mechanism

**Figure 3:**
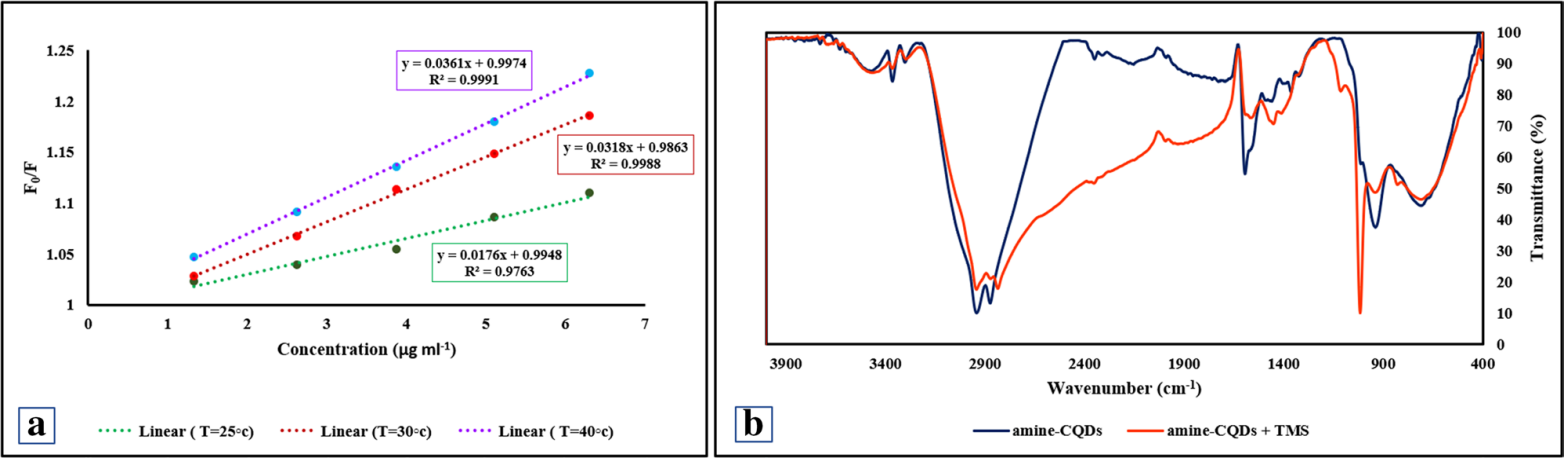
(**a**) The Stern–Volmer curves of TMS at the temperatures of 25 °C, 30 °C, and 40 °C, (**b**) FT-IR of amine-CQDs (navy color), and amine-CQDs with TMS (red color).

**Figure 4:**
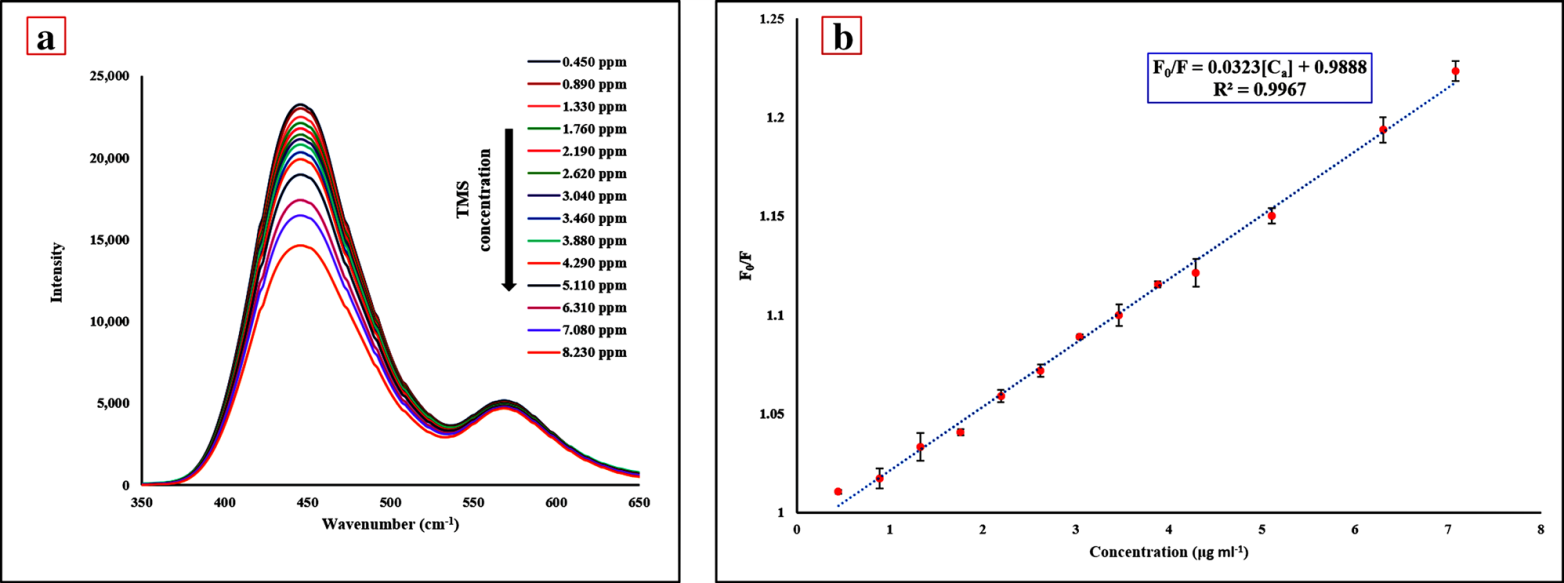
(**a**) FL emission spectra of CQD/RhB sensor in the presence of different concentrations of TMS, and (**b**) linear calibration plot in different concentrations of TMS (pH = 3, volume ratio = 0.2, and time = 15 min). Error bars represent the standard deviation from three repetitions.

To construe the process of TMS sensing by an amine-CQDs/RhB sensor, the mechanism between amine-CQDs and RhB, and amine-CQDs/RhB and were examined. According to Figure S5, the emission of conjugated amine-CQDs is reduced compared to free CQDs due to forming a new bond between amine-CQDs and RhB; this causes the Fluorescence resonance energy transfer (FRET) phenomenon to occur, so the emission intensity somewhat decreases^35^. According to Figure S2c, the absorption spectra of amine-CQDs and RhB do not overlap with each other. Therefore, due to the absorption spectral overlap with CQDs, TMS causes the phenomenon of FL quenching, while owing to the lack of spectral overlap with RhB, its emission does not change^36^. Photoinduced electron transfer (PET) can occur when the excited electron is transferred from CQDs (donor) to TMS (acceptor). Thus, for this reason, TMS connects to the carboxylic group of CQDs and creates a new interaction. In the following, we investigated the quenching behavior by way of the Stern–Volmer equation (Eq. 1), zeta potential analysis, and alterations of the FT-IR spectrum.

1 $$ {\text{F}}_{0} /{\text{F}} = {\text{K}}_{{{\text{sv}}}} \left[ {\text{Q}} \right] + 1 $$

where F_0_ and F are the FL intensities ratio of the RF sensor in the absence and presence of TMS, respectively. [Q] represents the concentration of quencher, ‘K_sv_’ is the Stern–Volmer quenching constant^37^. As shown in Fig. 3a, the value of F_0_/F was plotted for different concentrations of quencher at three temperatures. By increasing temperature, the slope of the curve that represents K_sv_ increased. So the FL quenching might have been happened due to dynamic quenching^38,39^. As it can be seen in Fig. 4a, by enhancing the concentration of TMS to this probe, the emission peak of RhB remains constant, while the emission intensity of CQDs reduces. So, TMS could have been detected by CQDs. To further investigate the sensing mechanism, the interaction between amine-CQDs and TMS was studied by zeta potential analysis. As shown in Figure S6a, free amine-CQDs have a zeta potential of − 45.8 mV, but the zeta potential of them in the presence of TMS makes a dramatic shift to − 33 mV, this reduces in the surface charge of the amine-CQDs after the adding TMS to the solution confirms that functional groups of amine-CQDs have reacted with TMS, so the FL signal reduces. The zeta potential of amine-CQDs in the presence of RhB is increased from − 45.85 to − 46.9 because the RhB has a negatively charged functional group; moreover, the zeta potential of the RF probe is reduced to − 43.75 in the presence of TMS, which indicates the formation of an electrostatic interaction (Figure S6b). Also, the interaction between amine-CQDs, RhB, and TMS was examined by FT-IR spectrum. In order to investigate the interaction between CQDs and RhB, the FT-IR spectrum of free and conjugated CQDs was taken (Figure S7a). The NH peak of free CQDS decreased at 1570 nm while the peak corresponding to C–N increased at 1650 in the conjugated CQDs spectrum. Therefore, a new interaction is formed between the methyl group of RhB and the amine group of CQDs, which is in accordance with the proposed in Reference article^40^. In order to examine the interaction between free CQDs and conjugated CQDs with TMS, the FT-IR analysis is performed. For free CQDs, when TMS was incubated in amine-CQDs solution, the band intensities of NH stretching vibration reduced to 3364 nm. Not only the absorption peak position of C=O has switched from 1594 to 1564 nm, but also the intensity has been lessened. In the FT-IR spectrum of CQDs + TMS, the absorption peak attributed to the N–O bond is increased in the 1016 nm region. This band is not observed in the FT-IR spectra of TMS and amine-CQDs. By reducing the intensity of the C=O peak and forming the C-O^−^ band, it can be turned out that a new electrostatic interaction has been established between the C-O^−^ group of amine-CQDs and the amine-group of TMS, which completes the sensing process (Fig. 3b). For conjugated CQDs similar to free CQDs, the peak intensity for the carboxylic acid group of conjugated CQDs decreased while the absorption peak for the N–O band increased (Figure S7b).

### Calibration and validation method

We analyzed the response of the RF probe at different concentrations of TMS under the optimal experimental conditions. By increasing the concentration of TMS to this probe, the signal intensity was reduced at λ = 442 nm (Fig. 4a). As shown in Fig. 4b, a good linear relationship could be observed between F_0_/F and the concentration of TMS in the range of 0.446–7.083 μg mL^−1^ (1.091–17.338 μM). The linear regression equation is F_0_/F = 0.0323[C_a_] + 0.9888 (C_a_ is the concentration of TMS), and the correlation coefficient is 0.9967 (n = 3). The detection limit (LOD) and quantitation limit (LOQ) of the proposed method for five tests were estimated to be 0.033 μg mL^−1^ (0.081 μM, 3S_b_/m) and 0.109 μg mL^−1^(0.267 μM, 10S_b_/m), respectively. In order to compare the LOD of the free amine-CQDs probe with the amine-CQDs/RhB probe, the calibration cure of free CQDs was plotted (Figure S8). The LOD of the free CQDs sensor was calculated as 2.033 μg mL^−1^ (4.976 μM). The comparison between the proposed RF probe and other reported methods to determine TMS is listed in Table 1. This method has a good linear range and a lower LOD than spectrometric methods; also, dual emission systems have a higher signal-to-noise ratio than single-signal systems, so they are more accurate and sensitive. This RF probe is cheaper, faster, and more environmentally friendly compared to chromatographic methods, as well as this method does not require a complicated apparatus.

**Table 1:** The comparison of the present study with other reported approaches for TMS detection.

Methods	Detection samples	Probe	LDR (μg mL^−1^)	LOD (μg mL^−1^)	LOQ (μg mL^−1^)	Refs.
EC	TMS	MWNTs–Nafion-modified GCE	0.133–445	0.044	–	^41^
RP-HPLC	TMS	-	10–50	0.495	0.461	^42^
LC–MS/MS	TMS	–	0.1–19.3 × 10^–3^	–	–	^43^
UV–VIS	TMS, SOL	–	15–70	1.250	3.800	^44^
FL	TMS, TOL, SOL	TMS/TOL and TMS/SOL	0.75–3.50	0.170	0.520	^45^
FL	TMS	Amine-CQDs/RhB	0.446–8.230	0.033	0.109	This work

**Figure 5:**
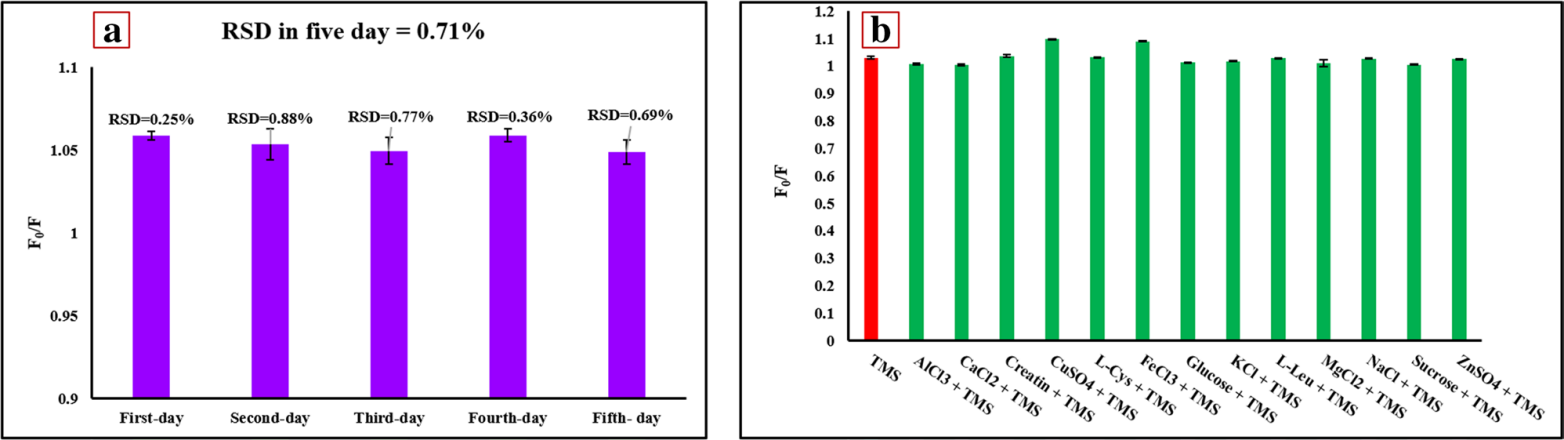
(**a**) Repeatability of RF sensor in five days (Five testes for each day), and (**b**) The influence of a few foreign species on the sensing system (C_TMS_, C_L-Cys_, C_L-Lus_, C_methyldopa_, C_levocarb_, and C_atenolol_ = 0.660 μg mL^−1^; C_tamoxifen_ = 1.2 μg mL^−1^; C_glucose_, C_sucaros_, C_Creatinin_, C_CuSO4,_ and C_FeCl3_ = 3.3 μg mL^−1^; C_NaCl_, C_KCL_, C_CaCl2_, C_MgCl2,_ and C_ZnSo4_ = 6.6 μg mL^−1^). Error bars represent the standard deviation from three repetitions of tests under optimal conditions (pH = 3, volumetric ratio = 0.2, and time = 15 min).

In order to evaluate the repeatability of the present recognizing system, we iterated one point by one operator at one place with the same working condition and the same measurement system. Twenty-five experiments were performed in five working days; in this way, five tests were done every day. Thus, as displayed in Fig. 5a, the relative standard deviation (RSD) for intra-day repeatability was calculated to be less than 0.88%, while the RSD for inter-day was computed at 0.71%.

### Selectivity

To validate the selectivity of the CQDs/RhB ratiometric probe towards TMS under the optimal conditions, we investigated the response of this sensor to some interfering substances (Al^3+^, Ca^+2^, Zn^2+^, Fe^3+^, Cu^2+^, Mg^2+^, K^+^, Na^+^, Glucose, Sucrose, L-Cys, L-Leu, and Creatin) in the presence of TMS. As illustrated in Fig. 5b, among interfering substances, except for copper, iron, Methyldopa, Levocarb, and Atenolol, all other species are less than 5% troublemakers on the sensing system. Both copper and iron have a 6.0% and 5.4% effect on the sensing process due to the formation of strong interaction with CQDs. Methyldopa, Levocarb, and Atenolol make about 8.2, 7.0, 5.0% effect, because these drugs have an amine group in their structure that can interact with CQDs. The acceptable outcome of selectivity analysis signifies that we can employ this ratiometric sensor to determine TMS in the actual condition.

### Analytical performance of the ratiometric sensor in biofluids

In order to demonstrate the practical application of the proposed method for TMS recognization in real samples, the experiments were carried out in human urea and serum (AL-Ghadir Hospital, Tehran, Iran). The recoveries of TMS in the human urea were obtained in the range of 94–106%, whereas the RSD range was between 1.1 and 5.7%. The recovery values in human serum were calculated from 92 to 102%, whereas RSD was acquired from 2.4 to 6%. The obtained results in Table 2 indicate that the RF probe is a reliable procedure for analyzing TMS in biofluids.

**Table 2:** Results for determination of TMS in real samples.

Sample	Real concentration	Spiked (μg mL^−1^)	Found ± SD (μg mL^−1^)	Recovery (%, n = 3)	RSD (%, n = 3)
Urea	ND	0.658 (1.610 μM)	0.702 ± 0.04 (1.735 ± 0.098 μM)	106.7	5.7
	ND	0.987 (2.440 μM)	1.022 ± 0.025 (2.526 ± 0.061 μM)	103.5	2.4
	ND	1.425 (3.523 μM)	1.356 ± 0.047 (3.353 ± 0.117 μM)	95.1	3.5
	ND	1.864 (4.608 μM)	1.753 ± 0.054 (4.336 ± 0.135 μM)	94.1	3.1
	ND	2.193 (5.421 μM)	2.210 ± 0.026 (5.463 ± 0.063 μM)	100.8	1.1
Serum	ND	0.767 (1.896 μM)	0.713 ± 0.025 (1.762 ± 0.061 μM)	92.9	3.5
	ND	0.987 (2.440 μM)	0.953 ± 0.023 (2.356 ± 0.056 μM)	96.6	2.4
	ND	1.096 (2.710 μM)	1.066 ± 0.049 (2.630 ± 0.120 μM)	97.2	4.5
	ND	1.864 (4.610 μM)	1.711 ± 0.046 (4.231 ± 0.115 μM)	92.0	2.7
	ND	1.974 (4.880 μM)	2.018 ± 0.121 (4.988 ± 0.300 μM)	102.2	6.0

## Discussion

Consequently, we developed an RF sensor to determine TMS, which this RF probe is constructed from two agents; amine-CQDs act as the reporter agent that turns specific interactions between the RF sensor and TMS into the recognizable signals, and RhB as a reference agent was utilized to remove interferences effects on sensing system. By adding TMS to this probe, the emission signal of amine-CQDs is quenched at 442 nm, while the emission signal related to RhB at 569 nm has remained constant. The parameters of pH, the volumetric ratio of CQDs to RhB, and interaction time for the RF sensor were optimized using the OVAT method. Therefore, This FL detection method does not require complicated and expensive apparatus, and it displayed high selectivity toward the detection of TMS over various species. According to the comparison made in Table 1, this method has the advantage of higher speed, lower cost, less complexity of the design system compared to reported literature. Besides, the practicability of this RF sensor was successfully validated by sensing TMS in human urea and blood serum samples, which showed satisfactory results that this method could be applied for rapid detection of TAM in biological fluids.

## Methods

### Reagents and materials

The active ingredient of Tamsulosin hydrochloride (TMS) was purchased from Tolid Daru Pharmaceutical Co (Tehran, Iran). The citric acid (CA), Ethylenediamine (EDA), Rhodamine B (RhB), l-Cysteine (l-Cys), l-leucine (l-Lue), Creatinin, Sucrose, Glucose, Iron (ɪɪɪ) chloride, Aluminum chloride, Calcium chloride, copper (II) sulfate, Magnesium chloride, Sodium chloride, Potassium chloride, Zinc sulfate, Acetic acid, Phosphoric acid, Boric acid, and Methanol (Me-OH) with the analytical grade were purchased from Merck Co. (Darmstadt, Germany). Deionized (DI) water with a resistivity of more than 18 MΩ has been used in the whole of the experiments.

### Apparatus

The fluorescence measurements were carried out by FL-Ar-2015 fluorescence spectrometer (teifsanje, Iran) and Cary Eclipse fluorescence spectrophotometer (Agilent, USA) with a 1.0 cm quartz cuvette. The Absorption measurements were performed through a T80 plus spectrophotometer (PG Instruments, Australia(. Transmission electron microscopy (TEM) images were obtained to analyze amine-CQDs morphology using a Philips-EM 208 s microscope (FEI Co., USA) at an accelerating voltage of 100 kv. Fourier transform infrared (FT-IR) spectra were acquired through Avatar (Thermo, USA), and ASCII (Perkin Elmer, UK) in the range of 400–4000 cm^−1^ by using KBr pellets. Zeta potential analysis was accomplished using an SZ100 (Horiba, Japan). EDS analysis of amine-CQDs was performed by a MIRA III FE-SEM (Tescan, Czech Republic(. The pHs value was measured and adjusted on a 691 pH meter (Metrohm Co., Swiss). Mixing the solutions was performed on a VORTEX Genius 3 (IKA, Germany).

### Synthesis of amine-CQDs

The amine-CQDs were constructed via a one-step hydrothermal method based on the previous work^46^. Briefly, 3.000 g CA and 15 mL EDA were dissolved in 45 mL DI water and then poured into a 100 mL Teflon-lined stainless-steel autoclave and treated with 200 °C for 5 h. The resulting brown solution was cooled down to room temperature, then the synthetic solution was filtered through a 0.22 μm membrane three times to remove the unreacted species, and finally, the prepared carbon dots were transferred into a dark container and kept at 4 °C.

### General procedure

**Figure 6:**
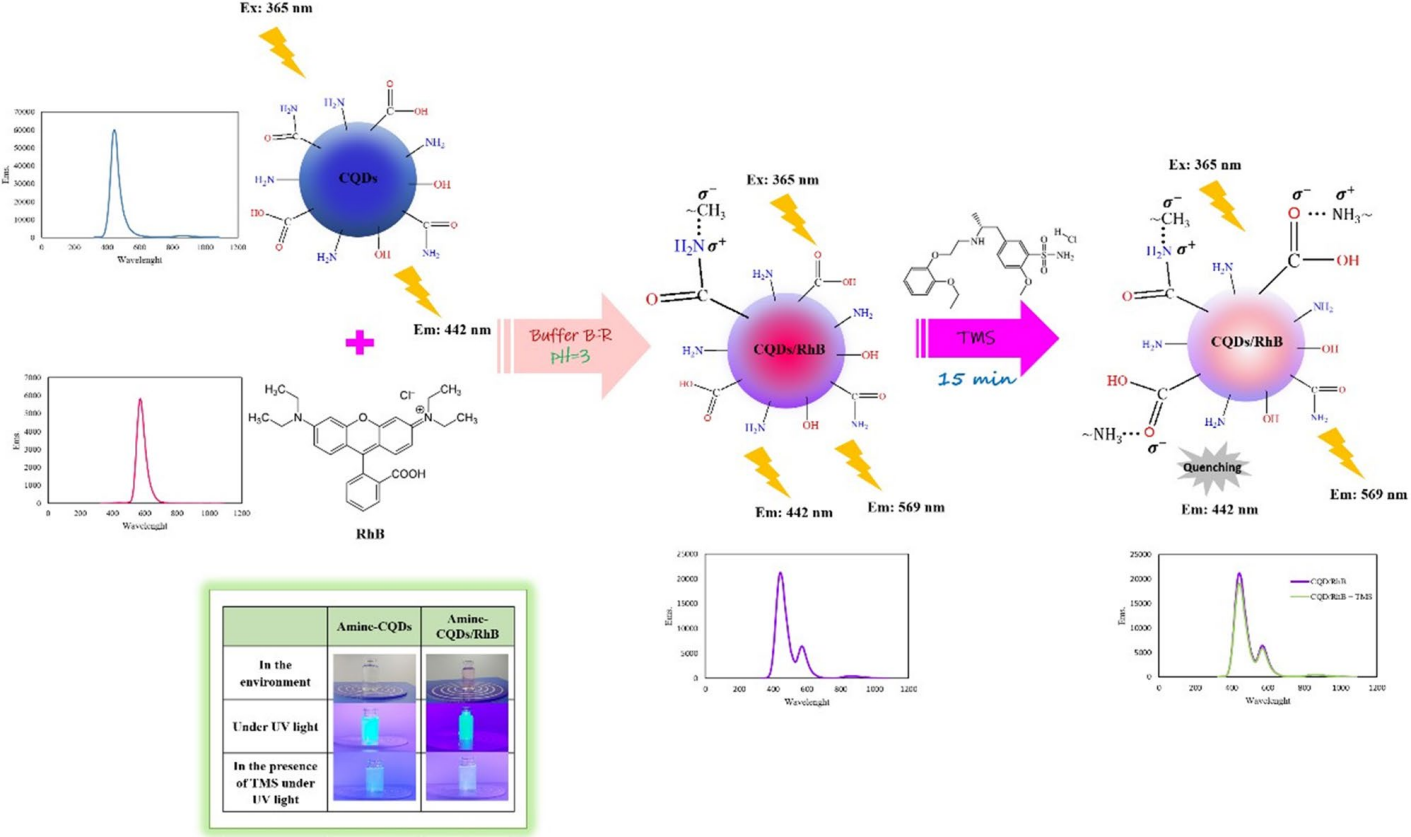
The process of sensing TMS using amine-CQDs/RhB RF probe (Drawn with Paint Application, Microsoft Windows 10, version 21H1).

Initially, 1 mL DI water and 200 μL Britton-Robinson (B-R) buffer (pH = 3) were transferred into the cuvette, and then 5 μL pure amine-CQDs and 25 μL rhodamine-B (20 μg mL^−1^) were injected into this solution. After recording the signal of the ratiometric probe, different concentrations of TMS were introduced into the above mixture by micropipette. Ultimately, after incubation for 15 min, the new signal was documented (Fig. 6).

### Sample treatment and ethical statement

To validate the reliability of the designed RF sensor under real conditions, human urea and blood serum were chosen as actual samples. Human blood serum and urea samples of healthy volunteers were collected in blood collection tubes and clean Falcons by trained staff of AL-Ghadir Hospital (Tehran, Iran). The volunteer gave informed consent with full cognizance of the aim of this project. All experiments and preparation were performed in compliance with the relevant laws and university guidelines as well as with the approval by the Ethical Committee of the Department of Chemistry, Iran University of Science and Technology, Tehran, Iran.

The urea sample was utilized freshly, but the serum sample was stored in the freezer at − 20 °C. The urea sample was centrifuged at 6000 rpm for 20 min, then 50 μL from that was poured separately into five 5 mL volumetric flasks, and different volumes of the standard solution of TMS were added to those flasks, and they were diluted with DI water and B-R buffer (pH = 3), so urea samples just diluted 100 times. The serum sample was thawed gently and was centrifuged at 10000 rpm for 10 min. 250 μL of serum sample was transferred to clean Falcon, 250 μL Me–OH and 5 mL buffer B–R (pH = 3) were added to the Falcon, it vortexed for 5 min, then was centrifuged at 9000 rpm for a quarter. 1 mL of the centrifuged solution was transferred individually into five 5 mL volumetric flasks, and various amounts of the standard solution of TMS were added to solutions, and dilution was done by DI water.

## Supplementary Information

Supplementary Information.

## Abbreviations

CQDs: Carbon quantum dots
RhB: Rhodamine B
TMS: Tamsulosin hydrochloride
BPH: Benign prostate hyperplastic
FRET: Fluorescence resonance energy transfer
PET: Photoinduced electron transfer
OVAT: One-variable-At-a-Time
DI: Deionized
B-R: Britton-Robinson
l-Cys: l-Cysteine
l-Leu: l-Leucine
EDA: Ethylendiamin
CA: Citric acid
Me-OH: Methanol
FL: Fluorescence
RF: Ratiometric fluorescence
CE: Capillary electrophoresis
HPLC: High-performance liquid chromatography
MS: Mass spectrometry
EDS: Energy-dispersive X-ray spectroscopy
UV–Vis: Ultraviolet
EC: Electrochemical
TEM: Transmission electron microscopy
FT-IR: Fourier transform infrared
LDR: Linear dynamic range
LOD: Detection limit
LOQ: Quantitation limit
ND: Not detected
MWNTs: Multiwalled carbon nanotubes
GCE: Glassy carbon electrode
