# Supplementary material for: Sensitive detection of tamsulosin hydrochloride based on dual-emission ratiometric fluorescence probe consisting of amine-carbon quantum dots and rhodamine B
Source: Sci Rep. 2021 Oct 21;11:20805. doi: 10.1038/s41598-021-00363-x (PMC8531343; doi:10.1038/s41598-021-00363-x)
Supplement: Supplementary file 1 — Supplementary Information. [file 41598_2021_363_MOESM1_ESM.pdf]

Supplementary Data

Sensitive detection of tamsulosin hydrochloride based on dual-emission ratiometric fluorescence probe consisting of amine-carbon quantum dots and rhodamine B

Aida Mousavi, Rouholah Zare-Dorabei *, Seyed Hossein Mosavi

Research Laboratory of Spectrometry & Micro and Nano Extraction, Department of Chemistry, Iran University of Science and Technology, Tehran 16846-13114, Iran

* **Corresponding author**: E-mail address: **zaredorabei@iust.ac.ir** (Rouholah Zare-Dorabei)

Tel: +98 21 77240646 & Fax: +98 21 77491204

Supplementary Caption

Figure S1 FT-IR spectra of (a) RhB, and (b) TMS

Figure S2 (a) the UV-Vis spectra of amine-CQDs and Rh-B, (b) the FL spectra of amine-CQDs in different excitation wavelength, and (c) the UV-Vis spectra of amine-CQDs/ Rh-B and TMS

Figure S3 (a) Absorption spectra, and (b)FL spectra of amine-CQDs to evaluate photostability

Figure S4 Photostability of amine-CQDs under UV irradiation for different times

Figure S5 the FL spectra of CQDs, Rh-B, and CQDs/RhB

Figure S6 Zeta potential analysis of (a) amine-CQDs in absence and presence of TMS (b) amine-CQDs/RhB in absence and presence of TMS

Figure S7 FT-IR spectra of (a) RhB, amine-CQDs, and amine-CQDs/RhB, and (b) amine-CQDs/RhB, and amine-CQDs/RhB + TMS

Figure S8 calibration cure of different concentrations of TMS, which is measured by free CQDs

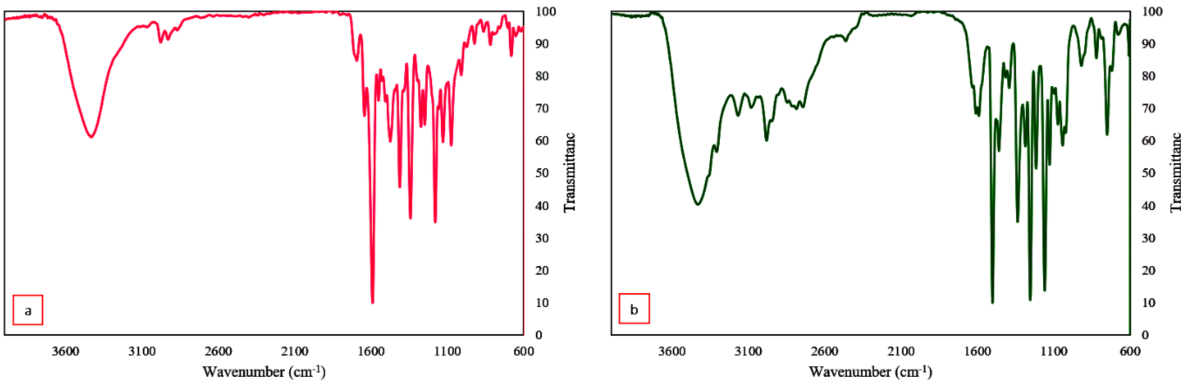

Figure S1 FT-IR spectra of (a) RhB, and (b) TMS

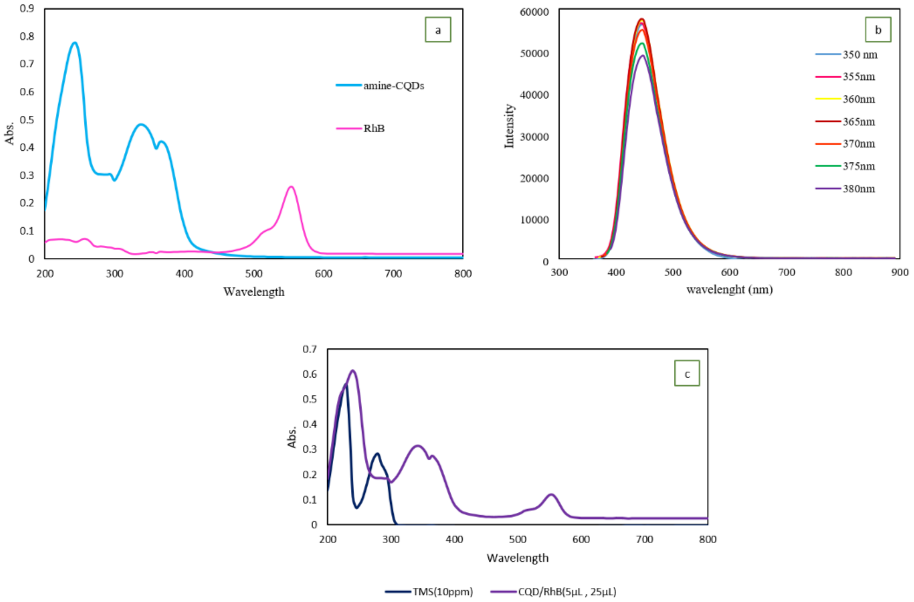

Figure S2 (a) the UV-Vis spectra of amine-CQDs and RhB, (b) the FL spectra of amine-CQDs in different excitation wavelength, and (c) the UV-Vis spectra of amine-CQDs/ RhB and TMS

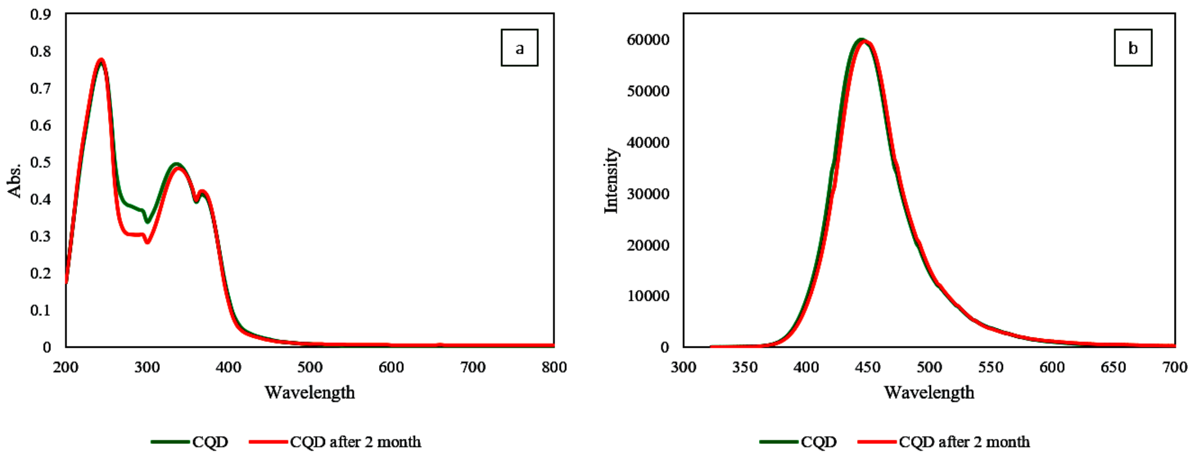

Figure S3 (a) Absorption spectra, and (b)FL spectra of amine-CQDs to evaluate photostability

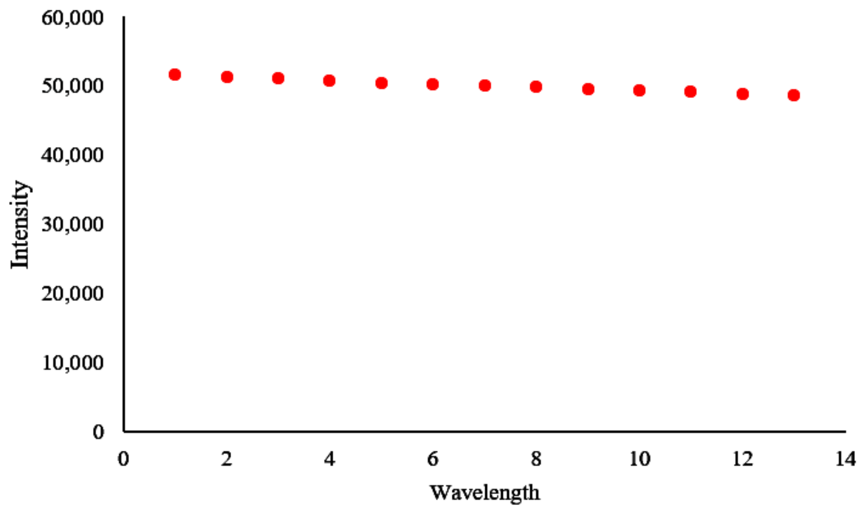

Figure S4 Photostability of amine-CQDs under UV irradiation for different times

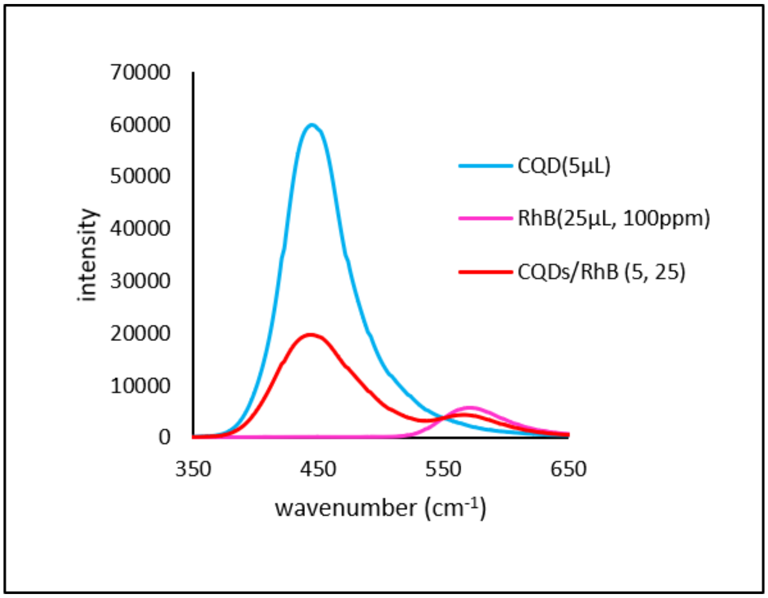

Figure S5 the FL spectra of CQDs, Rh-B, and CQDs/RhB

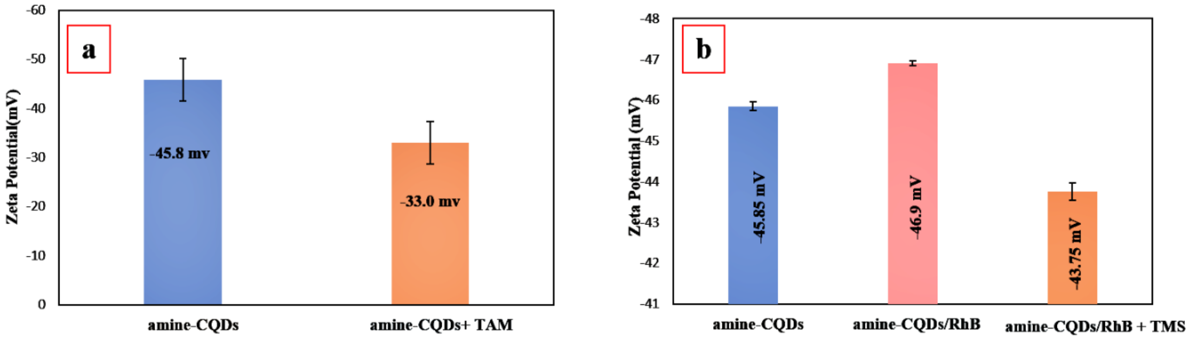

Figure S6 Zeta potential analysis of (a) amine-CQDs in absence and presence of TMS (b) amine-CQDs/RhB in absence and presence of TMS

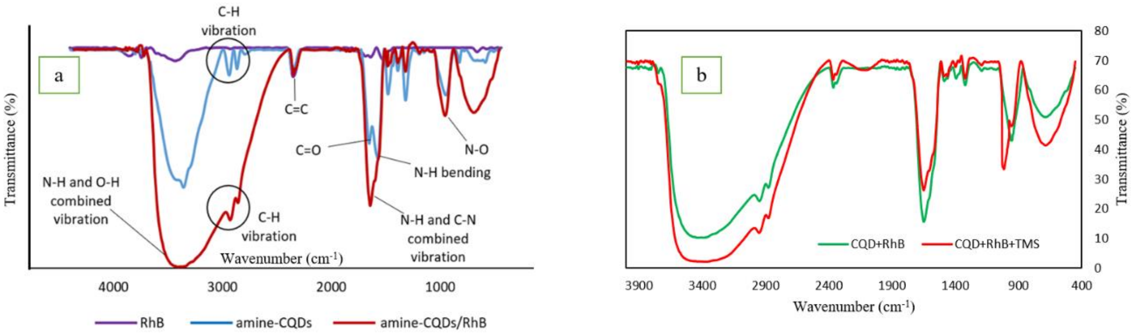

Figure S7 FT-IR spectra of (a) RhB, amine-CQDs, and amine-CQDs/RhB, and (b) amine-CQDs/RhB, and amine-CQDs/RhB + TMS

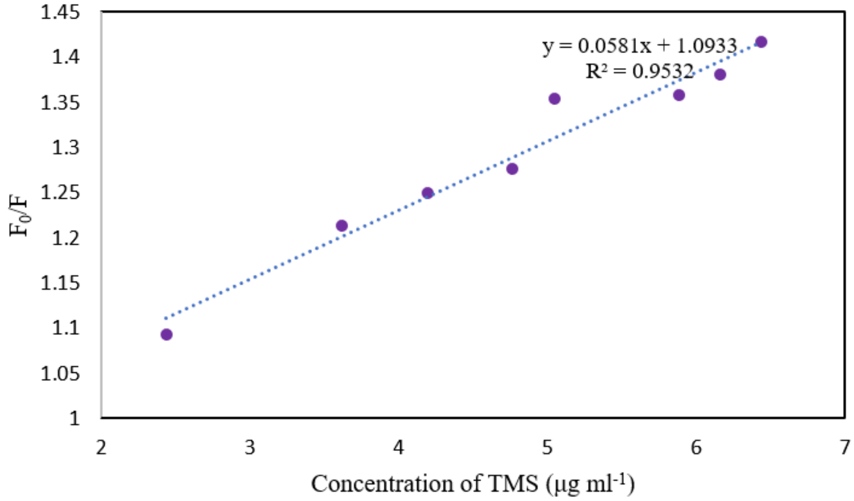

Figure S8 calibration cure of different concentrations of TMS, which is measured by free CQDs
